# Large language models for human-machine collaborative particle accelerator tuning through natural language

**DOI:** 10.1126/sciadv.adr4173

**Published:** 2025-01-01

**Authors:** Jan Kaiser, Anne Lauscher, Annika Eichler

**Affiliations:** ^1^ Deutsches Elektronen-Synchrotron DESY, Hamburg, Germany.; ^2^ Universität Hamburg, Hamburg, Germany.; ^3^ Hamburg University of Technology, 21073 Hamburg, Germany.

## Abstract

Autonomous tuning of particle accelerators is an active and challenging research field with the goal of enabling advanced accelerator technologies and cutting-edge high-impact applications, such as physics discovery, cancer research, and material sciences. A challenge with autonomous accelerator tuning remains that the most capable algorithms require experts in optimization and machine learning to implement them for every new tuning task. Here, we propose the use of large language models (LLMs) to tune particle accelerators. We demonstrate on a proof-of-principle example the ability of LLMs to tune an accelerator subsystem based on only a natural language prompt from the operator, and compare their performance to state-of-the-art optimization algorithms, such as Bayesian optimization and reinforcement learning–trained optimization. In doing so, we also show how LLMs can perform numerical optimization of a nonlinear real-world objective. Ultimately, this work represents another complex task that LLMs can solve and promises to help accelerate the deployment of autonomous tuning algorithms to day-to-day particle accelerator operations.

## Teaser

Large language models are capable of tuning particle accelerators after being queried only with a natural language prompt.

## INTRODUCTION

Particle accelerators are sophisticated machines designed to accelerate subatomic particles, such as electrons and protons, to extremely high speeds, often close to the speed of light. These devices play a crucial role in a variety of applications, ranging from fundamental research in physics to practical uses in medicine, such as cancer therapy and material science. As the demands from these diverse applications grow, there is an increasing need for advanced tuning and control methods to manage the complex dynamics of particle acceleration. Despite this, as a result of its complexity, the tuning of particle accelerators is to this day often done manually by experienced human operators. In this context, the emergence of autonomous tuning methods represents a substantial advancement. By leveraging methods from the fields of numerical optimization and machine learning (ML) (*1*–*3*), autonomous systems promise to speed up accelerator tuning procedures, reducing costs and minimizing downtime, while also enabling advanced operating modes for state-of-the-art measurements. Moreover, such methods enable a paradigm shift from actuator-driven accelerator operation, where human operators control actuator settings to achieve good measurement conditions, to specification-driven operation, where human operators determine the best conditions for experiments and autonomous agents ensure that these conditions are achieved. As such, autonomous particle accelerator tuning methods promise to not only improve the performance of accelerators on existing applications but also open up previously unexplored possibilities in scientific research and industrial applications, marking a transformative step in the field of particle acceleration.

However, implementing state-of-the-art accelerator tuning methods on new tuning tasks requires experts in two separate domains—accelerator physics and optimization—as well as substantial engineering effort to solve problems ranging from algorithm selection to objective function formulation. These challenges have so far slowed the adoption of advanced autonomous tuning algorithms to day-to-day accelerator operations.

In recent developments, large language models (LLMs), such as GPT 4 (*4*) and Llama 2 (*5*), have been demonstrated to be capable of solving complex tasks when prompted through natural language (*4*, *6*, *7*). The question arises whether LLMs can directly perform particle accelerator tuning when prompted by an accelerator expert describing the tuning goal. If capable, this would provide a more natural way of controlling autonomous tuning solutions through natural language, potentially enabling a more straightforward deployment of autonomous particle accelerator tuning solutions, and removing the requirement for optimization algorithm–specific expertise. Moreover, the ability of LLMs to explain their reasoning (*8*) could provide valuable insights into the complex dynamics of particle accelerators, potentially aiding human operators in understanding the tuning process. Last, the successful application of LLMs to particle accelerator tuning would also demonstrate the ability of LLMs to solve (multi-objective) numerical optimization problems, possibly opening up avenues for the application of LLMs to optimization tasks beyond particle accelerators.

Initial efforts toward autonomous accelerator tuning have investigated numerical methods such as Nelder-Mead simplex (*1*, *9*, *10*), robust conjugate direction search (RCDS) (*11*–*13*), extremum seeking (ES) (*14*), and genetic algorithms (*15*). These methods have since found adaptation in the day-to-day tuning of particle accelerator facilities (*16*–*18*). More recently, advanced methods like Bayesian optimization (BO) have found increased interest in the accelerator community (*2*) for their ability to use ML to learn a probabilistic surrogate model of the underlying objective function, enabling more sample-efficient tuning of high-dimensional and increasingly complex accelerator systems. Efforts are currently underway to lower the barrier of entry to these methods and increase their adoption in day-to-day accelerator operations (*19*). Moreover, the accelerator community is looking increasingly to ML methods to aid with the challenges of accelerator tuning (*20*). In particular, reinforcement learning (RL) has found adoption to accelerator control tasks (*21*, *22*). RL has also been successfully applied to so-called RL-trained optimization (RLO), where neural network (NN) policies are trained through optimizer learning (*23*–*26*) to be capable of sample-efficient accelerator tuning (*3*, *27*–*29*).

Most recently, LLMs have had a highly visible impact on the field of artificial intelligence (AI) and ML. Usually based on the transformer NN architecture, first introduced in (*30*), these models are trained to perform text completion such that they develop text understanding and text generation capabilities, which can be exploited to create chatbots. As such, state-of-the-art LLMs like GPT 4 (*4*) have been demonstrated to have not only impressive capabilities, such as text summarization, but also the ability to solve more complex tasks like coding and general problem solving. The field of LLMs is moving fast and seeing substantial investments. Despite their high training cost, many of these models have been released in a short time frame, both commercial and closed in nature, such as GPT 4 (*4*), Gemini (*31*), and Claude (*32*), but also numerous open-source (or more specifically open-weights) models, such as Llama (2) (*5*), Orca (2) (*33*, *34*), Starling-LM (*35*), and Mistral/Mixtral (*36*, *37*). Most of these are released in varying sizes with varying trade-offs between capabilities and computational efficiency.

The application of LLMs to optimization is less prominent in recent research than other applications. Naturally fitting the natural language processing (NLP) origins of LLMs, they have successfully been applied to optimizing prompts to LLM chatbots (*38*). In further work, LLMs have been used to find more effective algorithms than the state of the art to solve complex problems (*39*). Most similar to our work, the ability of LLMs to solve numerical optimization has been demonstrated on the simple task of linear regression in (*38*). A benchmark evaluating the performance of different LLMs on a game-playing task like those typically solved by training NN policies through RL is presented in (*7*).

In the context of particle accelerators, there exist ambitions to harness the NLP abilities of LLMs for various purposes. In (*40*), the authors demonstrate how to fine-tune an open-source LLM to be a particle accelerator domain expert using open-access scientific literature as training data, augmented by another LLM to generate question-answer pairs from research papers. The fine-tuned model, called PACuna, is shown to be more proficient in answering questions related to particle accelerators. In (*41*, *42*), the author demonstrates how off-the-shelf LLMs can be used as a general AI assistant for intelligent accelerator operations (GAIA), using the ReAct (*43*) prompting scheme to enable the LLM to intelligently trigger accelerator operation routines, automatically contact experts when needed, research questions in the facility’s logbook, provide the correct control system addresses for actuators and sensors of the accelerator, and write weekly shift reports.

Here, we introduce an approach to using LLMs for autonomous tuning of a particle accelerator. We answer whether current state-of-the-art LLMs are capable of solving particle accelerator tuning tasks, and whether they present a promising alternative to the current state of the art in particle accelerator tuning. To this end, we compare 14 different LLMs and 4 different prompts, and evaluate our LLM-based approach against other tuning algorithms, including RLO and BO.

## RESULTS

### Evaluation setup

For this work, we evaluate a total of 14 different LLMs, which are specified in detail below. We evaluate each of the LLMs with multiple different prompts on three different instances of the experimental area (EA) transverse beam parameter tuning task. The instances differ in the target beam parameters set by the human operator, the transverse misalignments of the quadrupole magnets and the diagnostic screen, the properties of the beam entering the EA section from upstream, and the initial magnet settings before the respective tuning algorithm has taken any action. We refer to these instances as trials. The transverse tuning task, where the goal is to set five magnet values to achieve a desired beam shape downstream, is known to be a nonlinear nonconvex optimization problem. The task and the EA section are described in detail in Methods. For each trial, we run each model and prompt three times with different random seeds to account for the stochasticity of the LLMs and some of the baseline algorithms.

Performance is evaluated in terms of the mean absolute error (MAE) between the measured beam parameters and the target beam parameters after 50 iterations [“Final beam difference (m)”]. This tests the ability of the models to find a good set of magnet settings. We further consider the normalized MAE improvement from the initial magnet settings to the final magnet settings found by the model, which tests the ability of the models to improve the beam parameters from the initial settings [“Normalized beam improvement (%)”]. Normalization by dividing the MAE improvement by the MAE with the initial magnet settings makes this metric less sensitive to the inherent variability and difficulty of different trials. Finally, we consider the normalized MAE over all interactions, which tests the ability of the models to find a good set of magnet settings quickly (“Number of successful steps”). Here, too, the impact of trial-to-trial variations is reduced by dividing by the accumulated MAE of keeping the magnet settings the same as the initial settings for 50 iterations. For all LLMs, we further consider the number of consecutive steps for which they are able to generate a parsable JSON output, which tests the models’ reliability in generating a valid output. LLMs are given a second chance in each sample, if they fail to generate a parsable JSON output on the first attempt.

The main goal of this work is not to determine whether LLMs are capable of outperforming the current state of the art in accelerator tuning algorithms. We expect that the current state of the art in accelerator tuning algorithms, such as RLO and BO, should clearly outperform LLMs. Instead, we hope to determine whether LLMs are capable of solving accelerator tuning (and by extension other complex optimization tasks) at all, and to what extent they can do so. We therefore also introduce three measures of “success,” where we consider a tuning run successful, if the final beam difference is at least 40 m improved over the initial beam difference before any tuning has taken place, with 40 m being twice the real-world measurement accuracy for beam parameters on the diagnostic screen. This means that runs are only considered successful if a clearly measurable improvement of the beam parameters has been achieved. A tuning algorithm is considered “outright successful” if it is able to achieve the success criteria in all nine evaluation runs. We consider a tuning algorithm as “partially successful” if it is able to achieve the success criterion in at least six of the nine evaluation runs. Partial success suggests that, while not perfectly reliable, successful runs are probably not coincidental. We further know that some trials can be harder to solve than others. As a third and weakest success criterion, we therefore consider a tuning algorithm as “single trial successful” if it is able to achieve the success criterion for each of the three runs of a single trial, suggesting that, while some trials may have been too difficult to solve, the model was able to reliably solve this one trial.

The 14 different LLMs that have been considered are Gemma 2B and Gemma 7B (*44*) (version 1.0); GPT 3.5 Turbo (checkpoint 0125) (*45*), GPT 4 (*4*) (checkpoint 0613), and GPT 4 Turbo (preview checkpoint 0125) (*46*); Llama 2 7B, Llama 2 13B, and Llama 2 70B (*5*), as well as the fine-tuned variants of Llama 2: Orca 2 7B and Orca 2 13B (*33*, *34*), and Vicuna 7B 16 K (*47*); Mistral 7B (version 0.2) (*36*) and Mixtral 8x7B (*37*); and Starling-LM 7B (beta) (*35*). In total, we evaluate four different templates for prompting the models (Tuning Prompt, Explained Prompt, Chain-of-Thought Prompt, and Optimization Prompt) to account for the sensitivity of the models regarding particular formulations of the same task (*48*). We explain the prompt templates in detail in the “Optimization scheme” section. For cost reasons and because the Explained Prompt and Chain-of-Thought Prompt are variations on the Tuning Prompt, the Explained Prompt and Optimization Prompt are evaluated with all models, while the Tuning Prompt and Chain-of-Thought Prompt are evaluated only with Gemma 2B, GPT 4 Turbo, and Mixtral 8x7B. This should provide a sense for how the additions of chain-of-thought and a task explanation affect performance with the representative LLMs while comprehensively comparing different LLMs and two very different prompts.

Prompt generation and response parsing are implemented using the LangChain (*49*) Python package, which provides a straightforward set of tools for constructing prompts, calling LLMs and parsing their responses. The open-weights LLMs used in this work are run using Ollama (*50*), while the OpenAI models are run through the OpenAI API. All models are run using their default temperature value, with T=0.7 for the OpenAI models and T=0.8 for all other models. Every LLM is prompted following its respective prompt format for system prompt, user prompt, and response. Orca 2 7B, Orca 2 13B, and Vicuna 7B 16 K are run with their default system prompts as listed in the Supplementary Materials. All other models are run without any system prompts as per their default configuration. A Gymnasium (*51*) environment of the EA transverse beam parameter tuning task using the Cheetah (*52*, *53*) beam dynamics simulator is used to evaluate the LLMs and baselines. The baselines BO, ES, and random search are implemented following (*54*). The RLO and do nothing baselines are implemented according to (*3*), using the trained policy model from that work.

**Fig. 1.**
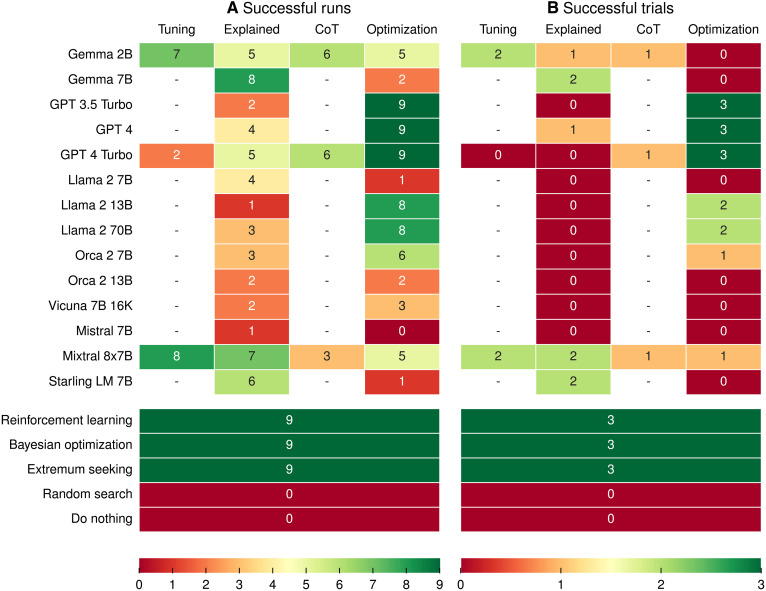
Number of successful runs and trials for each model and prompt. We define as success an improvement of at least 40 m on the beam differences when compared to the initial magnet settings. (**A**) Number of successful runs. (**B**) Number of wholly successful trials, i.e., trials where all three runs were successful.

**Fig. 2.**
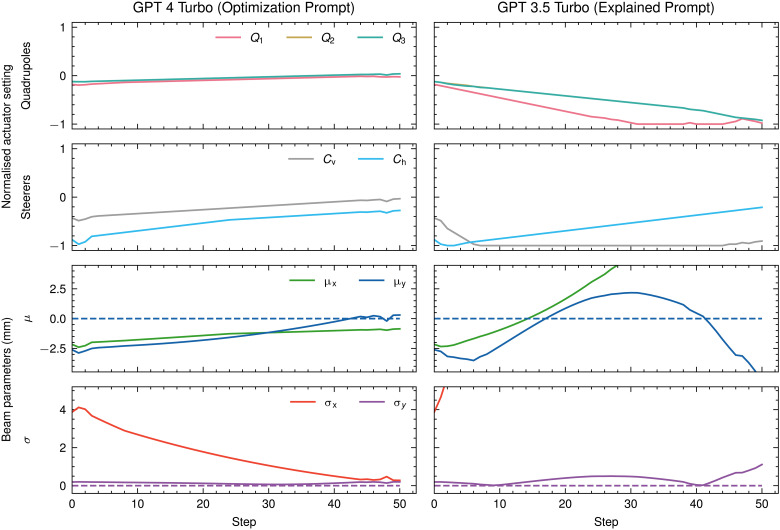
Magnet setting and beam parameter traces for a good and a bad tuning run by LLMs. Both runs used the same trial, where the target beam parameters are $\mu_{x}=\mu_{y}=\sigma_{x}=\sigma_{y}=0\;\text{mm}$.

The results of the evaluation in terms of the three previously defined metrics are shown in Table 1. The number of successful runs and wholly successful trials for each model and prompt are shown in Fig. 1 Two example tuning runs by a well-performing and a poorly performing model and prompt combination are shown in Fig. 2.

**Table 1.** Evaluation results. The metrics are given as mean ± SD. The best results for each metric are highlighted in bold. CoT, chain-of-thought.

	Final beam difference (m)		Normalized beam improvement (%)		Normalized integrated MAE (%)		Number of successful steps	
	Explained	Optimization	Explained	Optimization	Explained	Optimization	Explained	Optimization
Gemma 2B	1,665 ± 634	3,180 ± 5,187	11 ± 71	34 ± 171	115 ± 51	137 ± 88	23 ± 19	39 ± 14
Gemma 7B	1,651 ± 764	8,105 ± 12,933	−16 ± 11	284 ± 428	85 ± 10	247 ± 142	9 ± 0	29 ± 11
GPT 3.5 Turbo	11,593 ± 14,850	1,197 ± 771	397 ± 618	−36 ± 27	292 ± 245	78 ± 20	**50** ± 0	**50** ± 0
GPT 4	1,849 ± 1,445	1,213 ± 860	11 ± 73	−40 ± 25	98 ± 60	73 ± 20	**50** ± 0	**50** ± 0
GPT 4 Turbo	2,184 ± 1,879	**962** ± 740	20 ± 89	**−50** ± 28	107 ± 76	**67** ± 21	**50** ± 0	**50** ± 0
Llama 2 7B	1,432 ± 798	2,085 ± 779	−12 ± 55	15 ± 27	94 ± 15	106 ± 15	8 ± 6	3 ± 4
Llama 2 13B	1,936 ± 772	1,507 ± 821	5 ± 26	−22 ± 23	101 ± 10	85 ± 21	0 ± 1	13 ± 20
Llama 2 70B	1,947 ± 964	1,539 ± 942	10 ± 42	−21 ± 27	107 ± 37	92 ± 16	**50** ± 0	**50** ± 0
Orca 2 7B	2,149 ± 1,222	1,377 ± 855	17 ± 42	−23 ± 37	122 ± 65	93 ± 17	4 ± 3	4 ± 7
Orca 2 13B	1,634 ± 875	3,232 ± 3,684	−13 ± 24	77 ± 170	88 ± 18	142 ± 92	1 ± 2	3 ± 2
Vicuna 7B 16 K	4,756 ± 5,332	4,331 ± 3,829	184 ± 320	320 ± 580	189 ± 137	234 ± 236	34 ± 7	48 ± 7
Mistral 7B	2,551 ± 1,233	19,653 ± 23,427	48 ± 57	803 ± 869	121 ± 40	1,574 ± 1,513	**50** ± 0	30 ± 22
Mixtral 8x7B	1,606 ± 1,158	1,901 ± 1,192	**−24** ± 27	−14 ± 31	**76** ± 26	101 ± 26	**50** ± 0	45 ± 14
Starling LM 7B	**1,401** ± 449	7,659 ± 7,249	1 ± 69	363 ± 521	98 ± 60	324 ± 252	36 ± 15	19 ± 16
	Tuning	CoT	Tuning	CoT	Tuning	CoT	Tuning	CoT
Gemma 2B	1,452 ± 525	**955** ± 702	−14 ± 46	**−40** ± 49	97 ± 43	87 ± 60	10 ± 1	**50** ± 0
GPT 4 Turbo	2,647 ± 1,827	1,337 ± 813	45 ± 81	−23 ± 45	119 ± 64	**70** ± 25	**50** ± 0	**50** ± 0
Mixtral 8x7B	**1,321** ± 771	1,775 ± 926	**−29** ± 23	−8 ± 17	**71** ± 22	95 ± 19	**50** ± 0	**50** ± 0
	Baselines							
RLO	**16** ± 17		**−99** ± 1		**3** ± 1		–	
BO	100 ± 26		−93 ± 6		31 ± 23		–	
Extremum seeking	457 ± 267		−71 ± 19		35 ± 17		–	
Random search	7,677 ± 3,830		487 ± 588		647 ± 476		–	
Do nothing	1,967 ± 903		0 ± 0		100 ± 0		–	

### Performance

We find that the state-of-the-art tuning algorithms RLO and BO, as well as ES, all achieve the strictest success criterion of outright success in all nine evaluation runs. Of the LLM prompt combinations evaluated, GPT 3.5 Turbo, GPT 4, and GPT 4 Turbo in combination with the Optimization Prompt also achieve outright success in all nine evaluation runs, with GPT 4 Turbo with the Optimization Prompt also being the best-performing LLM prompt combination in all evaluated metrics. In addition, a further 10 LLM prompt combinations achieve partial success, with Llama 2 13B, Llama 2 70B, and Orca 2 7B doing so with the Optimization Prompt; Gemma 7B, Mixtral 8x7B, and Starling LM 7B achieving partial success with the Explained Prompt; Gemma 2B and Mixtral 8x7B achieving partial success with the Tuning Prompt; and Gemma 2B and GPT 4 Turbo achieving partial success with the Chain-of-Thought Prompt. Overall, Mixtral 8x7B is the best-performing model with the Explained Prompt, but is outperformed by Starling LM 7B on the Final Beam Difference metric. With the Tuning Prompt, Mixtral 8x7B performs best of the three evaluated models, while Gemma 2B is the best-performing model with the Chain-of-Thought Prompt. All models that achieve partial success also achieve single trial success in at least one trial, demonstrating that they are able to solve some trials reliably. A further six LLM prompt combinations achieve single trial success: Gemma 2B and GPT 4 with the Explained Prompt, Mixtral 8x7B with the Optimization Prompt, and Mixtral 8x7B with the Chain-of-Thought Prompt. In total, of the 34 LLM prompt combinations tried, 18 achieve at least one success criterion. Of 14 LLMs evaluated, 10 achieve at least one success criterion with at least one prompt. This demonstrates that LLMs can be used to solve accelerator tuning tasks.

However, these results also show that LLMs are not yet competitive with the state-of-the-art accelerator tuning algorithms. The best-performing LLM prompt combination, GPT 4 Turbo with the Optimization Prompt, achieves an average normalized beam improvement of −50%. This is a good result, but it is also a notably worse result than the −99% and −93% achieved by RLO and BO, respectively. A similar trend can be observed in how fast algorithms are able to find a good set of magnet settings. GPT 4 Turbo with the Optimization Prompt achieves an average normalized integrated MAE of 67%, which is an order of magnitude worse than the 3% achieved by RLO. However, it is only about two times worse than BO and ES.

### Choosing a prompt

The results show that the performance of LLMs is highly dependent on the specific model and prompt used. While 18 of the 34 LLM prompt combinations tried achieve at least one success criterion, the remaining 16 do not achieve any. Similarly, 4 of the evaluated LLMs do not achieve any success criterion with any of the prompts they were tested on. We observe that in general, the Optimization Prompt performs best in our evaluations. Outright success was only achieved with the Optimization Prompt, and at least one success criterion was achieved by seven LLMs when using the Optimization Prompt, while only five LLMs achieve at least one success criterion with the Explained Prompt. The Optimization Prompt is also used in the best-performing LLM prompt combination with GPT 4 Turbo. That, however, does not mean that the Optimization Prompt is always the better choice. Some models perform better with one of the other prompts. Gemma 7B, Mixtral 8x7B, and Starling LM 7B, for example, all achieve partial success with the Explained Prompt, but only single trial success or no success criterion at all with the Optimization Prompt. Similarly, Gemma 2B and Mixtral 8x7B achieve their best results with the Tuning Prompt. We conclude that the choice of prompt must be made on a model-by-model basis.

It is also worth noting that adding explanations to the prompts about how the magnets work, or adding a chain-of-thought to the prompts, does not always lead to the expected improvements. Of the three models evaluated with all four prompts, only GPT 4 Turbo improves with the addition of explanations. However, this is with GPT 4 Turbo generally performing badly on any of the three variants of the Tuning Prompt, generally performing better with the Optimization Prompt. Gemma 2B and Mixtral 8x7B, on the other hand, perform worse when the explanations are added. A possible explanation for this observation is that, rather than helping the model understand the tuning task, the length of the explanations makes it harder for the LLM to retrieve relevant information, such as specific past samples or the target beam parameters, from the prompt. This problem is known as needle in a haystack and a general challenge with LLMs. Chain-of-thought prompting seems to improve performance over the Explained Prompt with Gemma 2B and GPT 4 Turbo, but has an adverse effect on the performance of Mixtral 8x7B. These results also suggest that intuitive improvements of the prompt are not always beneficial, and reinforce the conclusion that the choice of prompt must be made on a model-by-model basis.

In designing the presented LLM tuning solution, we found that aside from getting LLMs to successfully tune the particle accelerator, another difficulty is to get them to output the magnet settings in a parsable JSON format. This is why LLMs are given a second chance in each sample, if the parsing of their response fails on the first attempt. Nevertheless, some models fail on the second attempt as well, at which point we consider the tuning run terminated. We can therefore take the number of performed iterations (excluding second attempts) as an indicator of a model’s ability to produce a valid JSON output when provided with one of our prompts. Note that excluding second attempts, this is the number of interactions with the accelerator, not the number of times the LLM was prompted. The observed number of iterations for the nine evaluation runs of each model and prompt are shown in Table 1. We observe that many models, often those achieving good tuning results, have a high number of successful steps, with models like those by OpenAI and Llama 70B always achieving the maximum of 50 successful steps, regardless of the prompt used. Other models, such as both Orca 2 and the smallest variant of Llama 2, consistently struggle to produce a valid JSON output, with the number of successful steps being very low for either prompt. While in most cases it appears that the ability to generate valid JSON responses depends mostly on the LLM used, we also observe that the choice of prompt can have an impact in a few cases, with the difference being especially pronounced for the Gemma models, which achieve a higher number of successful steps with the Optimization Prompt than with the Explained Prompt. It does not appear as though one prompt is generally better than the other in terms of the number of successful steps. Furthermore, the nature of different invalid responses varies greatly. In some cases, the mistakes are so minor that human experts might fail to spot them, for example, when a trailing comma is added to the last JSON property. This is not allowed in JSON syntax and causes the parser to fail. Another failure case is related to chain-of-thought. For example, Orca 2—a model specifically trained to respond with chain-of-thought—often outputs an explanation of a strategy to solve the optimization problem rather than the next magnet settings requested in the prompt. Last, but certainly not least, some models fail to output a coherent response altogether, with responses being nonsensical, for example, starting the response with an invalid continuation of a JSON object and then continuing with multiple valid JSON objects even though only a single one was requested. In this case, both the invalid JSON object and the ambiguity about which JSON object should be parsed make the response invalid. Examples of these three described failure modes are given in Supplementary Text.

### Choosing a model

**Fig. 3.**
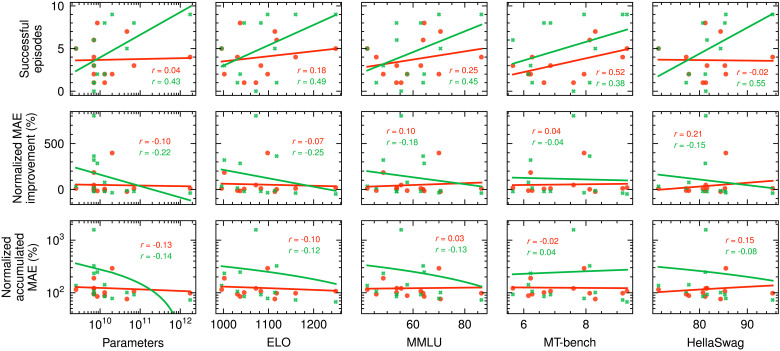
Number of successful tuning runs, average normalized MAE improvement, and average normalized accumulated MAE for each LLM with respect to its size, LMSYS Chatbot Arena ELO rating, MMLU score, MT-bench score, and HellaSwag score. Results for the Explained Prompt are shown in red, and results for the Optimization Prompt are shown in green. Linear fits are shown for the presented data. Pearson correlation coefficients *r* are provided for the shown samples in the respective subplots. We expect the number of successful episodes to increase and the other two metrics to decrease if model size or high benchmark scores improve the ability of LLMs to solve the investigated particle accelerator tuning task.

It is well known that some LLMs generally perform better than others. Often, an LLM’s capabilities are correlated with the number of parameters it has. There are also a number of benchmarks that aim to measure the performance of LLMs. These include the LMSYS Chatbot Arena ELO rating (*47*), the MT-bench score (*47*), the Massive Multitask Language Understanding (MMLU) score (*55*), and the HellaSwag score (*56*). When plotting the number of successful episodes, normalized beam improvement, and normalized integrated MAE over number of model parameters and benchmark scores in Fig. 3, we find that there typically is at least a weak correlation, in particular when considering results using the Optimization Prompt, where we measure Pearson correlation coefficients as high as 0.55 between the number of successful episodes and the HellaSwag benchmark results. The Pearson correlation coefficients are listed in Fig. 3. This finding suggests that the size and benchmark performance of an LLM can to some extent serve as an indicator for its performance on particle accelerator tuning and general numerical optimization tasks. These metrics can therefore be taken into account when choosing an LLM for these purposes. This observation further implies that, as increasingly well-performing general purpose LLMs are released, we can probably expect to see better performance on accelerator tuning and numerical optimization tasks.

### Resource requirements

Apart from LLMs’ ability to solve a given task, it is also important to consider the fact that LLMs are usually very resource intensive to run. The open-weights models used in this work are run on four NVIDIA A100 graphics processing units (GPUs) with 80 GB of memory each. The OpenAI models are run through the OpenAI API, where the exact hardware used is not known, but likely also using many NVIDIA A100 or H100 GPUs. In contrast, the state-of-the-art accelerator tuning algorithms RLO and BO can easily be run on laptop central processing unit (CPU), specifically an Apple M1 Pro system on a chip (SOC) for the results presented in this work. An average inference takes less than 200 s for RLO and around 700 ms for BO. In contrast, the fastest open-weights LLM was Gemma 2B on the Tuning Prompt with an average inference time of 700 ms, while the slowest was Orca 2 13B with 30 s on the Explained Prompt. Orca 2 inference is particularly slow because its chain-of-thought responses are long. We see similarly long inference times at 28 s when prompting GPT 4 Turbo with the Chain-of-Thought Prompt. Otherwise, the OpenAI models achieved between 1 s for GPT 3.5 Turbo on the Optimization Prompt and 4 s for GPT 4 on the Explained Prompt. A large open-weights model like Llama 2 70B achieved an average inference time of 7 s on the Optimization Prompt in our evaluations.

Such large resource demands usually induce high cost. While the actual cost of running LLMs on our own GPUs is difficult to estimate, the cost of running the OpenAI models through the OpenAI API as of 10 April 2024 is around USD 5.35 for one tuning run with GPT 4 and the Explained Prompt, and USD 2.98 for GPT 4 with the Optimization Prompt. GPT 4 Turbo costs less at around USD 1.81 for a tuning run using the Explained Prompt and USD 0.74 for the Optimization Prompt. GPT 3.5 Turbo was the cheapest, with API costs of around USD 0.09 and USD 0.05 for the Explained and Optimization Prompt, respectively. When using prompts that are likely to include more than a magnet setting JSON in the response, such as the Chain-of-Thought Prompt, the cost of running an optimization with GPT 4 Turbo increases to USD 2.63.

Considering the large amount of compute resources these models require, we must also consider their energy consumption and associated environmental impact. In (*57*), the authors find that GPT 3 consumes 500 ml of water for 10 to 50 responses. For the 50 responses in one evaluated tuning run, this comes out to 0.5 to 2.5 liters of water. While the authors do not mention the number of tokens assumed for a response, we can safely assume that these numbers are a lower bound for the much more resource-intensive GPT 4 and GPT 4 Turbo models used in this work. To estimate the CO_2_ emissions associated with using these models for particle accelerator tuning, we can consider Mixtral 8x7B as a representative model somewhat of average size. Taking the average inference time of 6 s per step with the Optimization Prompt, this model uses a total of 300 s of GPU time on four A100 GPUs. The energy consumption of a single A100 GPU is quoted as 250 W (*58*), i.e., 1 kW for all four GPUs, giving a total energy consumption of 83 W h for one tuning run. This is about the same amount of energy it takes to run a modern fridge for 11 h (*59*) or drive a modern electric vehicle for 0.5 km (*60*), and results in CO_2_ emissions of about 36 g (*61*). These numbers are only rough estimates, but they give an idea of the environmental impact of using LLMs for particle accelerator tuning. Generally, these should be lower for the smaller open-weights models, but higher for larger models like GPT 4 and GPT 4 Turbo. Note that none of the given numbers consider the environmental impact of training these models, which is substantial. However, as the models are already trained for other purposes and available, we do not take this into account in our evaluation.

To put these resource requirements into context, it is worth considering the amount of tuning that is typically required during accelerator operations. At the European X-ray Free Electron Laser (XFEL) facility at DESY, 2248 hours were spent on accelerator tuning in 2022 (*62*), and 1920 hours in 2023 (*63*). Under the assumption that LLM-based tuning takes the same amount of time as the tuning procedures currently in place, with half of that time spent on LLM inference, an average inference time per step of 4 s, and 1.66 W h of energy consumed on each inference step, this would amount around 1.6 MW h of energy consumption and 700 kg of CO_2_ emissions per year. For comparison, the total annual energy consumption of the ARES and European XFEL facilities is around 0.3 and 62 GW h, respectively (*64*). That means that the energy consumption of LLM-based tuning would amount to around 0.003% to 0.5% of the total energy consumption of such facilities. What is more, this is about half the energy consumption of the average German household per year (*65*), and the CO_2_ emissions are about 30% of the annual CO_2_ emissions of a mid-sized car (*66*).

## DISCUSSION

Here, we demonstrated that LLMs can be used to solve accelerator tuning tasks and, by extension, general numerical optimization tasks. However, considering a combination of 14 different open-weights and commercial LLMs and 4 different prompts, we find that only 18 of the 34 LLM prompt combinations can successfully achieve an improvement on the transverse beam parameter tuning task considered in this work. We conclude that, while it is generally possible to use LLMs for accelerator tuning, the choice of model and prompt is crucial. Comparing to state-of-the-art accelerator tuning algorithms, we further find that LLMs are not yet competitive with RLO and BO. The best-performing LLM prompt combination, GPT 4 Turbo with the Optimization Prompt, achieves an average normalized beam improvement of −50%, which is only about half as good as the −99% and −93% achieved by RLO and BO, respectively. While not achieving competitive performance, LLMs also incur high computational costs, leading to long inference times, high monetary costs, and notable environmental impact.

Despite these clear disadvantages that mean LLMs are not yet a viable alternative to state-of-the-art accelerator tuning algorithms, our results present an intriguing proof of concept. The field of LLMs is rapidly evolving, with ever more capable models being released on a near-daily basis. We have shown that more capable models generally perform better on accelerator tuning tasks, meaning that the inevitable progress in the field of LLMs will also lead to better performance on accelerator tuning tasks. Ultimately such development could make the intuitive deployment of autonomous accelerator tuning solutions through natural language a feasible option.

In the near future, we expect that, instead of being used as a replacement for state-of-the-art accelerator tuning algorithms, LLMs will find applications as copilots to human particle accelerator operators. Here, they can provide a natural language interface to various tasks related to accelerator operations, such as retrieving information from logbooks, generating reports, or diagnosing the accelerator’s state from large amounts of diagnostic measurements. Such efforts are already underway (*40*–*42*). In continuation of this work, we believe that LLMs could also be used to coordinate state-of-the-art accelerator tuning algorithms, such as RLO and BO, in a federated setting, deciding or helping the operator decide which part of the accelerator to tune next, using which algorithm and with which desired outcome. What is more, LLMs could also be used to assist human operators in the deployment of state-of-the-art tuning algorithms, for example, by proposing Xopt (*19*) configurations, or objective functions and suitable actuators in response to natural language prompts about the desired outcome. In the longer term, the approach of letting LLMs perform tuning directly may be improved by using a ReAct prompting scheme (*43*) or using LLMs to check if the magnet settings proposed algorithms like RLO and BO are sensible in a setup similar to (*67*, *68*).

## METHODS

### Particle accelerator tuning task

For the purpose of this work, we consider a specific particle accelerator tuning task, namely, the transverse beam parameter tuning in the EA section of the accelerator research experiment at SINBAD (ARES) linear particle accelerator (*69*, *70*) at DESY in Hamburg, Germany. This task has been chosen as it is a well-defined and well-understood task in the accelerator community, and has been extensively studied in the context of autonomous accelerator tuning (*3*, *54*, *71*, *72*). At the same time, the task is complex enough to be difficult to solve manually and can provide a meaningful benchmark for the capabilities of LLMs in accelerator tuning, yet simple enough such that solutions can still be easily understood and evaluated. Solving it would provide a valuable streamlining of accelerator operations because similar transverse tuning tasks can be found at most accelerator facilities and have to be regularly performed during everyday operations.

**Fig. 4.**
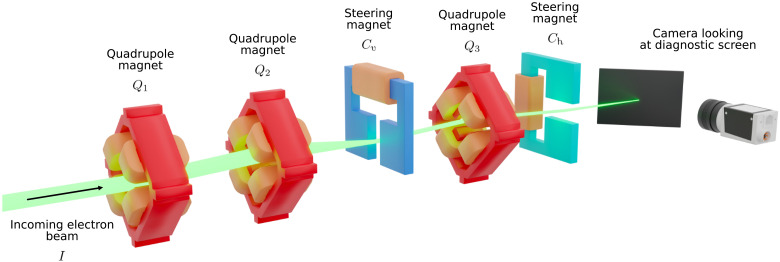
Schematic of the EA section of the ARES linear particle accelerator. Quadrupole magnets are shown in red; the vertical and horizontal dipole are shown in blue and turquoise, respectively. The electron beam is shown as a green envelop passing through the magnets and onto the screen at the end of the experimental area.

The EA section is primarily made up of five magnets as shown in Fig. 4. Three of these magnets are quadrupole magnets, $Q_{1}$, $Q_{2}$, and $Q_{3}$, which are used to focus the beam, and two are dipole magnets, $C_{v}$ and $C_{h}$, which are used to bend the beam, one in the vertical plane and one in the horizontal plane. Here, we control the focusing strength *k* of the quadrupole magnets in m^−2^ and the angle α by which the dipole magnets deflect particles in mrad. Note that turning up the strength of a quadrupole magnet will focus the beam in the horizontal plane and defocus it in the vertical plane, while turning down the strength will have the opposite effect. Increasing the steering angle of the vertical steering magnet will steer the beam upward, while decreasing the angle will steer the beam downward. The horizontal steering magnet works similarly, steering the beam to the right when the angle is increased and to the left when the angle is decreased. What is more, if the beam is off-center as it passes through the quadrupole magnet, the beam will additionally experience an angular deflection as it would with a dipole magnet. Any tuning task involving quadrupoles is therefore complex. The magnets are arranged in the order $\left(Q_{1},Q_{2},C_{v},Q_{3},C_{h}\right)$. At the end of the EA section, there is a diagnostic screen station. At the screen station, a screen made of a scintillating material is inserted into the beam pipe. When electrons pass through the screen, light is emitted, which is then captured by a camera and used to measure a transverse projection of the beam. Transverse beam parameters of beam position $\mu_{x,y}$ and beam size $\sigma_{x,y}$ can then be computed from the screen image by fitting a 2D Gaussian distribution. The goal of the tuning task is to find a set of magnet settings $\left(k_{Q_{1}},k_{Q_{2}},\alpha_{C_{v}},k_{Q_{3}},\alpha_{C_{h}}\right)$ that minimize the difference between the measured beam parameters $\left(\mu_{x},\sigma_{y},\mu_{y},\sigma_{y}\right)$ and some target beam parameters $\left(\mu \prime_{x},\sigma \prime_{y},\mu \prime_{y},\sigma \prime_{y}\right)$ set by the human operator.

We consider different instances of this tuning task, which we call trials. Trials differ in three aspects: The quadrupole magnets and the diagnostic screen have transverse misalignments m. These affect the dipole effect the quadrupoles have on the beam and shift the beam position measured on the screen. The misalignments are usually not known. Additionally, the incoming beam I entering the EA from upstream varies from day to day and between working points. It is difficult to measure the incoming beam, and therefore, it is also considered unknown. Finally, the target beam parameters $\left(\mu \prime_{x},\sigma \prime_{y},\mu \prime_{y},\sigma \prime_{y}\right)$ may differ from one tuning run to the next as they can be requested by the operator based on the experimental requirements.

### Optimization scheme

**Fig. 5.**
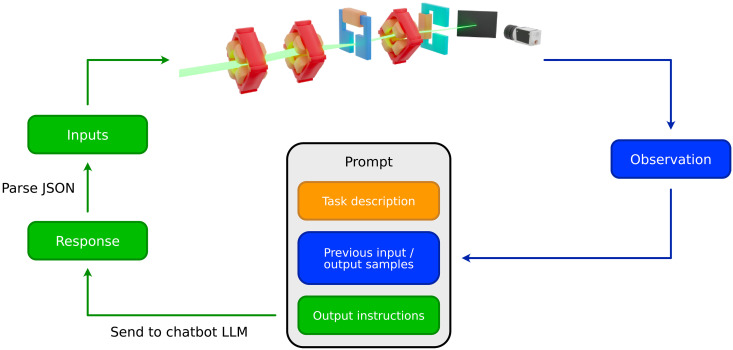
Flowchart of the optimization scheme used to tune particle accelerators using LLMs. The prompt is made up for three components: Task description, list of previous input and output samples, and instructions for what to output and how to format the output. The prompt is then sent to the LLM, which generates a response. The response is parsed into values that can be input into the tuning or optimization task. A measurement or objective value from the task is then inserted into the previous samples along with its corresponding input, and the loop is repeated.

**Fig. 6.**
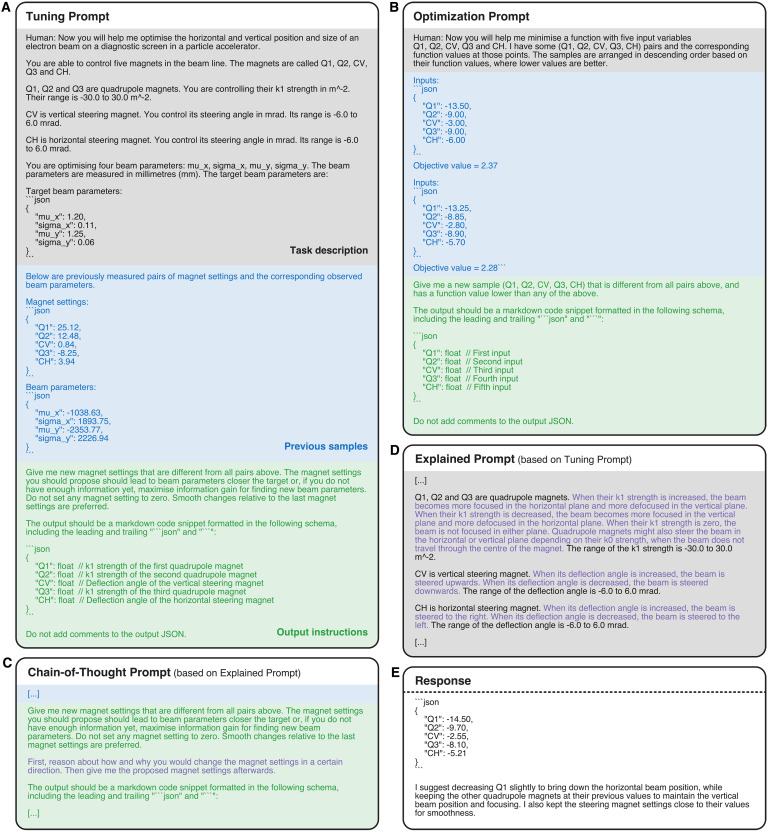
Examples of the evaluated prompts. Different parts of the prompts are highlighted in different colors, with the task description in gray, the input-output pairs in blue, and the request for the next input parameters and output instructions in green. Purple text highlights changes compared to the original prompt. (**A**) Tuning Prompt. (**B**) Optimization Prompt. (**C**) Modification of the Explained Prompt to make the Chain-of-Thought Prompt. (**D**) Modification of the Tuning Prompt to make the Explained Prompt. (**E**) Example of a response.

We consider an iterative optimization scheme for accelerator tuning, where the state of the accelerator is observed, and then the tuning algorithm chooses new actuator settings based on the current and all past states from the tuning run. This process is repeated either for a fixed number of iterations or until some termination criterion is met. For an LLM to act as the tuning or optimization algorithm, a prompting scheme needs to be devised. In our approach, we consider the use of a chatbot LLM, where the user can provide a question or command to the LLM and the LLM will respond with an answer. Our optimization scheme using LLMs extends on the approach for linear regression presented in (*38*) and is shown in Fig. 5. In the prompt to the LLM, the user provides a description of the optimization task that the LLM should solve. This is followed by a list of input and output pairs from previous optimization steps. After this list, the user asks for the next set of input parameters that help optimize the objective function and gives the LLM instructions on how these should be formatted such that the user can parse the output from text to numerical values. This prompt is then sent to the LLM, which responds with the next set of input parameters that should be used to optimize the objective function, and potentially also an explanation of why these parameters were chosen. The response should look similar to the one listed in Fig. 6. It is then parsed, and the input parameters are used to evaluate the objective function. The output of this evaluation is then added to the list along with its corresponding input parameters, and the process is repeated.

Prompt engineering is a crucial part of using LLMs and can notably affect the performance of the model. Because of the variability in the performance of different prompts and the difficulty of finding the best prompt, we evaluate the ability of LLMs to solve the accelerator tuning task using four different prompts: Tuning Prompt (see the “Tuning Prompt” section), Explained Prompt (see the “Explained Prompt” section), Chain-of-Thought Prompt (see the “Chain-of-Thought Prompt” section), and Optimization Prompt (see the “Optimization Prompt” section). All prompts follow the general prompting scheme described above, of task description, input-output pairs, request for next input parameters, and instructions on how to format the output. The prompts used in this work differ mainly in the task description and the outputs of the previous optimization steps.

#### Tuning Prompt

The Tuning Prompt is the most straightforward and intuitive prompt used in this work. It describes the task of tuning the transverse beam parameters in the EA section and the goal of achieving some target beam parameters on the diagnostic screen such that the LLM is aware of the fact it is tuning a particle accelerator. The input-output pairs are the magnet settings and the corresponding measured beam parameters. This prompt assumes that the LLM has some understanding of particle accelerators and understands, for example, what a quadrupole magnet is and how it affects the beam. An example of the Tuning Prompt is provided in Fig. 6, where the task description is highlighted in gray, the input-output pairs are in blue, and the request for the next input parameters and output instructions is in green.

Note that the choice was made to provide previously observed magnet settings and beam parameters formatted as a markdown JSON snippet. We found that if these are provided as a simple textual list of property names and their values, the LLMs would often output the next magnet settings in the same format instead of the requested JSON format. By providing the examples in the same format as we desire for the output, the parsing reliability of the LLM is increased substantially.

#### Explained Prompt

The Explained Prompt is mostly the same as the Tuning Prompt, but includes additional explanations of how each of the magnets affects the beam. This is done because accelerator physics is a complex and niche field, which is unlikely to have been widely covered in the training data of most general-purpose LLMs. The explanations are generally kept on a high level, similar to how one might explain the task to a new accelerator operator to give them an intuition of how the magnets affect the beam on the diagnostic screen. In Fig. 6, an example of the Explained Prompt is provided with the explanations highlighted in violet.

#### Chain-of-Thought Prompt

Chain-of-thought prompting (*8*) is a technique where the user asks the LLM to explain its reasoning before it gives its answer. This was found to generally improve the quality of the answers given by LLMs, especially in the case of logical reasoning tasks. Note that it is important to have the explanation before the answer, as otherwise the model will phrase the explanation in support of the already given and potentially incorrect answer, thereby negating the benefit of chain-of-thought prompting. In the Chain-of-Thought Prompt, we add a request to the prompt whereby the users asks the LLM to explain its reasoning before it gives the next set of input parameters. Otherwise, the Chain-of-Thought Prompt is the same as the Explained Prompt. We give an example of the Chain-of-Thought Prompt in Fig. 6, where the request for chain-of-thought reasoning is highlighted in violet.

#### Optimization Prompt

The Optimization Prompt phrases the task as a numerical optimization problem instead of a particle accelerator tuning task. This means that the model is completely unaware that it is tuning a particle accelerator. Numerical optimization tasks are more generic than particle accelerator tuning tasks and therefore expected to be more present in the training data used to train LLMs, meaning that models are likely to be more familiar with them. However, this also means that the model is given no information about the topology of the objective function, which makes the optimization problem harder to solve. The objective function is therefore a black box to the model. The input-output pairs are the magnet settings and a corresponding single scalar objective value computed from the beam parameters as

$$\text{objective}=|\mu_{x}-\mu_{x\prime }|+|\mu_{y}-\mu_{y\prime }|+|\sigma_{x}-\sigma_{x\prime }|+|\sigma_{y}-\sigma_{y\prime }|$$ (1)

We list an example of the Optimization Prompt in Fig. 6, where the task description is highlighted in gray, the input-output pairs are in blue, and the request for the next input parameters and output instructions is in green.
