# Supplementary material for: Large language models for human-machine collaborative particle accelerator tuning through natural language
Source: Sci Adv. 2025 Jan 1;11(1):eadr4173. doi: 10.1126/sciadv.adr4173 (PMC11691695; doi:10.1126/sciadv.adr4173)
Supplement: Supplementary file 1 — Supplementary Text [file sciadv.adr4173_sm.pdf]

Science Advances
AAAS

Supplementary Materials for

Large language models for human-machine collaborative particle accelerator tuning through natural language

Jan Kaiser *et al.*

Corresponding author: Jan Kaiser, jan.kaiser@desy.de

Sci. Adv. **11**, eadr4173 (2025)
DOI: 10.1126/sciadv.adr4173

This PDF file includes:

Supplementary Text

System Prompts

Orca 2 7B and Orca 2 13B:

```
You are Orca, an AI language model created by Microsoft.  You
are a cautious assistant.  You carefully follow instructions.
You are helpful and harmless and you follow ethical guidelines
and promote positive behavior.
```

Vicuna 7B 16K:

```
A chat between a curious user and an artificial intelligence
assistant.  The assistant gives helpful, detailed, and polite
answers to the user's questions.
```

Failed Responses

Incoherent response generated by Gemma 2B with the Explained Prompt:

````
sigma_y":  320.27 } ``` Magnet settings:  ```json { "Q1":
-14.33, "Q2":  -1.63, "CV":  0.02, "Q3":  15.98, "CH":  2.95 }
``` Beam parameters:  ```json { "mu_x":  1232.76, "sigma_x":
138.02, "mu_y":  1013.97, "sigma_y":  1370.42 } ``` Magnet
settings:  ```json { "Q1":  0.00, "Q2":  0.00, "CV":  1.26, "Q3":
0.00, "CH":  0.00 } ``` Beam parameters:  ```json { "mu_x":
-183.38, "sigma_x":  46.87, "mu_y":  2400.06, "sigma_y":  648.22
} ```
````

Long chain-of-thought response without properly formatted set of next magnet settings generated by Orca 2 13B with the Explained Prompt:

```
To help you optimise the beam parameters, I will use a genetic
algorithm that iteratively mutates and crosses the existing
magnet settings to produce new ones.  The fitness function of
the algorithm is based on the difference between the target and
```
```

the observed beam parameters, weighted by their standard deviations. The goal is to minimise this difference while satisfying the constraint that no magnet setting can be zero. Here are the steps I will follow:

1. Initialize a population of 20 random magnet settings, each represented as a JSON object. 2. Evaluate the fitness of each magnet setting by calculating the sum of the squared differences between the target and the observed beam parameters, multiplied by their standard deviations. Normalize this sum to have a mean of zero and a standard deviation of one. This is called the information gain. 3. Select the top 10% of the population based on their information gain as the parents for the next generation. Randomly select two of them as the donors for crossbreeding. 4. For each donor, generate a random magnet setting that differs from it by one unit in either direction (either positive or negative) with a probability of 0.5. This is called the child A. 5. For the other two donors, generate a random magnet setting that differs from them by two units in either direction (either positive or negative) with a probability of 0.25. This is called the child B. If there are less than four donors left, only one child B will be generated. 6. Append the child A and child B to the population, replacing the corresponding donor if there is no overlap. This way, each donor can contribute at most two children to the next generation. 7. Repeat steps 2 to 6 until the population size reaches 50 or the information gain does not improve by more than 0.01 for three consecutive generations. If either condition is met, output the best 20 magnet settings as the final answer. Otherwise, output a message indicating that the algorithm has not converged and needs more iterations.

Here is an example of how the algorithm works on the first generation:

Population: ["Q1": -5.83, "Q2": 7.67, "CV": -4.09, "Q3": -7.53, "CH": -4.17, ...]

Fitness (information gain): [-0.04, 0.03, 0.05, 0.08, 0.07]

Select top 10%: ["Q1": -5.83, "Q2": 7.67, "CV": -4.09, "Q3": -7.53, "CH": -4.17, ...]

```
Donors for crossbreeding: ["Q1": -5.83, "Q2": 7.67, "CV":
-4.09, "Q3": -7.53, "CH": -4.17, ...]

Child A: "Q1": -6.83, "Q2": 8.67, "CV": -3.09, "Q3": -8.53,
"CH": -3.17

Child B: "Q1": -4.83, "Q2": 9.67, "CV": -1.09, "Q3": -9.53,
"CH": -1.17

Population: ["Q1": -5.83, "Q2": 7.67, "CV": -4.09, "Q3":
-7.53, "CH": -4.17, "Q1": -6.83, "Q2": 8.67, "CV": -3.09,
"Q3": -8.53, "CH": -3.17, "Q1": -4.83, "Q2": 9.67, "CV":
-1.09, "Q3": -9.53, "CH": -1.17]

Fitness (information gain): [-0.04, 0.04, 0.04, 0.08, 0.06]

### Final answer: The algorithm has not converged and needs
more iterations.
```

JSON for next magnet settings generated with trailing comma not permitted in JSON syntax by Llama 2 7B with the Explained Prompt:

```
```json { "Q1": -10.5, "Q2": -8.25, "CV": -3.26, "Q3": -8.75,
"CH": -4.5, } ```
```
```
